# First person – Henrike Berns

**DOI:** 10.1242/dmm.052408

**Published:** 2025-05-14

## Abstract

First Person is a series of interviews with the first authors of a selection of papers published in Disease Models & Mechanisms, helping researchers promote themselves alongside their papers. Henrike Berns is first author on ‘ A homozygous human *WNT11* variant is associated with laterality, heart and renal defects’, published in DMM. Henrike is a medical student at the Faculty of Medicine, University of Freiburg, Freiburg, Germany. She conducted the research described in this article while an MD student in Dr Peter Walentek's lab at the University of Freiburg, Freiburg, Germany, investigating *WNT11* variants and their implications in human development, using the *Xenopus* model organism.


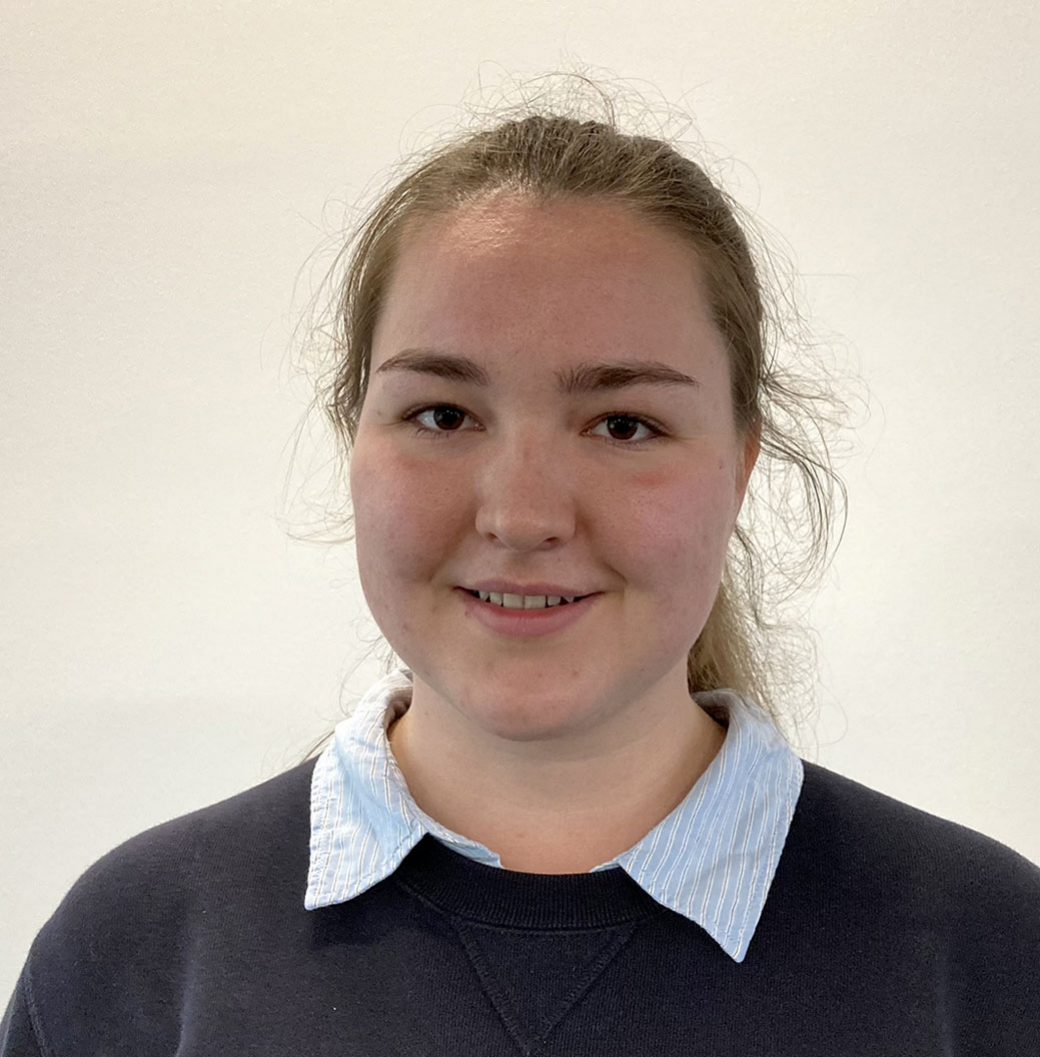


**Henrike Berns**

**Who or what inspired you to become a scientist?**

I have always been very interested in science and mathematics, and from an early age started bombarding my parents with questions. When I was 12, my family moved to India, where I attended an International School with a clear focus on scientific subjects. This had a big impact on me. In India, and later at another school in the UK, I had very dedicated and inspiring teachers, some of whom had previously worked in research themselves. They introduced me to scientific work and encouraged me to pursue my own first research projects. I then decided to study medicine, which I enjoyed; nevertheless, I was curious to learn more about the research behind medical knowledge and decisions. Therefore, I took a year off from my medical studies and joined the lab of Dr Peter Walentek to learn about basic biomedical research and pursue an exciting MD thesis project.

**What is the main question or challenge in disease biology you are addressing in this paper? How did you go about investigating your question or challenge?**

We have identified a novel *WNT11* variant in a patient family with laterality, heart and renal defects. Homozygous *WNT11* variants had not been described in humans before. We therefore wanted to explore this interesting variant and figure out its function, using the *Xenopus* (frog) model. During this investigation, I found that the *WNT11* variant encoded a ligand with reduced stability. This was startling because the variant, which results in a C-terminally truncated ligand, has very similar length to several *Xenopus wnt11b* constructs that had previously been shown to have dominant-negative activity in *Xenopus* research. I therefore decided to genetically clone several constructs of *WNT11* and *wnt11b* and compare their activities. Here, I found that only slight alterations in ligand length and composition of the C-terminal end of truncated WNT11 ligands dramatically alter their stability and function.

We describe the first patient case that has been identified with a homozygous *WNT11* variant.

**How would you explain the main findings of your paper to non-scientific family and friends?**

We describe the first patient case that has been identified with a homozygous *WNT11* variant. The patient showed mirror-inverted body organs, small kidneys and a complex heart defect. These phenotypic deviations had previously been described in animal research using mice and frogs. The *WNT11* variant leads to a shortened protein, which we have found to be instable and therefore less active. With description and functional examination of the human patient's variant, we could draw the link between existing results of animal research and human development and health. Furthermore, we explored how small changes in the length of the WNT11 protein have big effects on its functional activities. For this, we cut the gene at different positions, creating genetic constructs that had only slight differences in length. We injected these constructs into *Xenopus* embryos and looked at different read-outs, especially regarding the signalling cascade involved in the breakage of left-right body symmetry.

**What are the potential implications of these results for disease biology and the possible impact on patients?**

The patient case described in the paper, and correlations with existing research with *Wnt11* loss-of-function mice and *wnt11b* loss-of-function frogs, suggest that biallelic hypomorph *WNT11* activity results in laterality, kidney and heart defects in humans. Therefore, WNT11 dysfunction should be considered in patients with a combination of these defects. Furthermore, we found that small modulations to the length and composition of WNT11 ligands alter their activities significantly. Thus, even slight changes in genetic variants can severely impact the patient's outcome.

**Figure:**
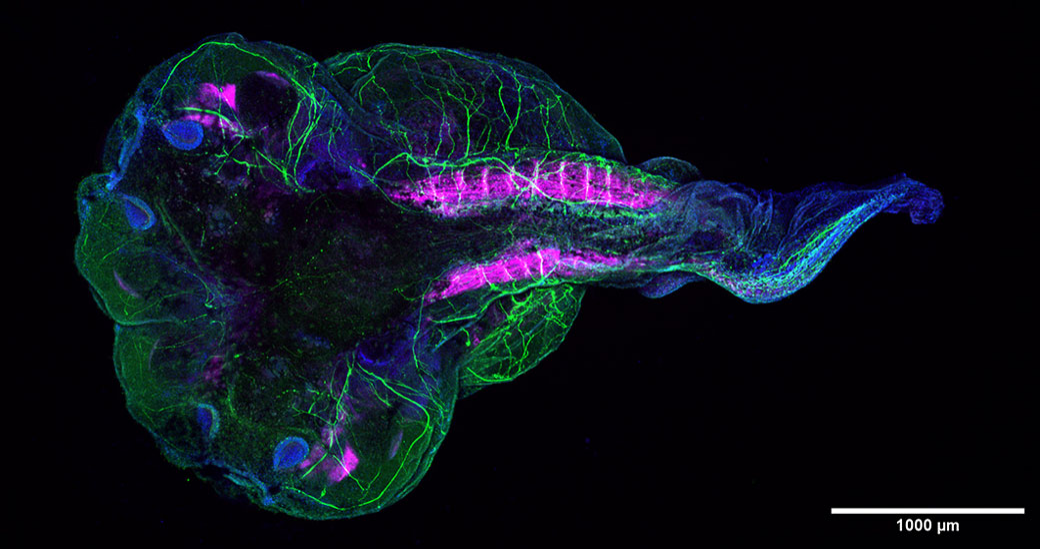
**Two-headed *Xenopus laevis* embryo with a short and twisted tail.** Immunofluorescent staining for neurofilament-associated antigen (by 3A10; green), filamentous actin (by phalloidin; pink) and nuclei (by DAPI; blue).

**Why did you choose DMM for your paper?**

The content of our paper is very translational, combining several research fields, including genetics, developmental biology, structural biology, rare disease, internal medicine and paediatrics. Moreover, the research was performed using the *Xenopus* model organism and serves as an example how *Xenopus* can be used to study human disease. We therefore felt that DMM suited the scope of the paper well. Additionally, it was important to us to publish Open Access with a not-for-profit publisher.

**Given your current role, what challenges do you face and what changes could improve the professional lives of other scientists in this role?**

It is a challenge for physicians to combine the treatment and care of patients and research activity. Nevertheless, I believe that progress in medicine can be made in particular with the help of people who are experienced in both fields. Studying medicine, I realised that there was not a lot of space for research activity in the relatively tight schedule of medical school. I was privileged to get the chance to work in the lab of Dr Peter Walentek and receive a scholarship with a suitable 1-year framework programme for medical scientists from the medical faculty of Freiburg that enabled me to get an insight into basic research. Fortunately, an increasing number of opportunities, such as clinician scientist programs, provide a structured framework for physicians to engage in research.

**What's next for you?**

I am currently finishing my medical studies, spending the ‘practical year’ (last year of medical school) in various hospitals gaining experience in several specialised departments. Meanwhile, I finished my MD thesis project in the lab of Dr Peter Walentek and hope to defend my thesis this summer. After completing medical school, the next step is training to become a medical specialist. I have yet to concretise the direction of my specialisation and would be happy if the option to conduct research arises again in the course of my career.

**Tell us something interesting about yourself that wouldn't be on your CV**

I have a collection of more than 100 tourist magnets! I love to travel and explore new countries and cultures and have collected magnets as a memory from many places since I was 10 years old. My family and I have always travelled extensively, and, in my youth, we spent 6 years abroad as an expat family. My curiosity about everything new and unfamiliar continues to this day, whether it concerns research, diseases and their treatment, or travelling.
